# Clinical description of two cases of Cowden syndrome and the implication regarding thyroid cancer

**DOI:** 10.1530/EDM-23-0105

**Published:** 2024-03-20

**Authors:** Stephanie Patrick, Deirdre James

**Affiliations:** 1 Division of Endocrinology, Department of Medicine, The University of Tennessee, Memphis, Tennessee, USA

**Keywords:** Paediatric, Adolescent/young adult, Female, Male, Black - African, White, United States, Skin, Thyroid, Thyroid, Genetics and mutation, Unique/unexpected symptoms or presentations of a disease, March, 2024

## Abstract

**Summary:**

Thyroid cancer is one of the most common manifestations of Cowden syndrome, yet the syndrome is rare. The incidence of Cowden syndrome is 1 in 200,000. The diagnosis can be made clinically when patients present with a combination of symptoms such as mucocutaneous lesions with a strong personal or family history of thyroid, breast, endometrial, and colorectal cancer. A high index of suspicion is required to provide a clinical diagnosis utilizing major and minor criteria. Once a clinical diagnosis is made, genetic testing for a PTEN mutation, a tumor suppressor gene, is recommended. Cancer surveillance should be performed for those with positive genetic testing as well as those with negative genetic testing who still meet clinical diagnostic criteria. We present two cases of Cowden syndrome: one case involving an increasing number of thyroid nodules in a patient with known Cowden syndrome and another patient with a strong family history of cancer, personal history of follicular thyroid cancer, and numerous colonic polyps on screening colonoscopy. These cases demonstrate how early diagnosis of Cowden syndrome can help detect early cancer in both the patient and affected relatives.

**Learning points:**

- Diagnosing Cowden syndrome helps pre-risk stratification for early cancer screening.
- The diagnosis of Cowden syndrome can be made with a combination of major and minor criteria: any two major criteria with or without a minor criterion; one major and one minor criterion; or three minor criteria.
- Patients who meet the diagnostic criteria for Cowden syndrome should undergo genetic screening.

## Background

Cowden syndrome (CS) is an autosomal dominant condition associated with a combination of mucocutaneous lesions along with an increased risk of thyroid, endometrial, colorectal cancer, and breast cancer (1). CS is associated with the phosphatase and tensin homolog (PTEN) tumor suppressor gene mutation, which leads to errors in cell proliferation (1, 2). The prevalence of CS is 1 in 200,000, making the diagnosis quite rare (1). Patients with CS have an increased lifetime risk of thyroid cancer by 33% compared to the average population (3). The following will be a report of two rare cases of CS which initially presented with thyroid cancer.

## Case presentation

We present two cases of CS. First, an 18-year-old female (patient no. 1) with autism and Hashimoto’s thyroiditis presented with increasing neck fullness and a various number of lipomas throughout her body. She denied any temperature intolerance, menstrual irregularities, weight changes, palpitations, or bowel irregularities. She underwent annual thyroid ultrasound screenings due to her mother’s history of CS and recent neck fullness. The thyroid ultrasound revealed two newly formed thyroid nodules since her last thyroid ultrasound.

Secondly, a 51-year-old male (patient 2) with a past medical history of follicular thyroid cancer status post radiation and thyroidectomy in 2019 presented to establish care for post-thyroidectomy management. He reported increasing skin papules on his face, neck, and back that he wished to have removed. He had an extensive family history of breast, colon, and renal cancer. He denied any constipation or diarrhea. He reported compliance with his levothyroxine 175 µg daily oral dose.

## Investigation

**Figure 1:**
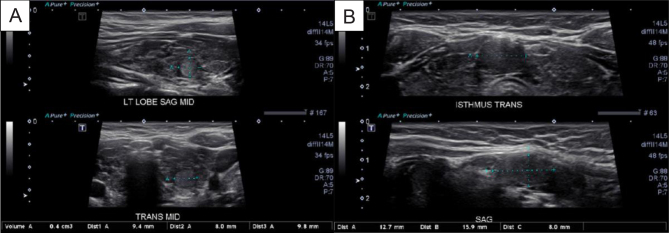
Thyroid ultrasound of two newly present thyroid nodules, a left thyroid nodule (A) and an isthmus nodule (B), that were not present 3 years beforehand.

Patient 1’s thyroid function tests showed a thyrotropin of 2.4 μIU/mL (0.450–4.50 μIU/mL) (2.4 mIU/L) and free thyroxine of 1.65 ng/dL (0.82–1.77 ng/dL) (11.45 pmol/L) while on levothyroxine 100 µg daily. Thyroid ultrasound revealed two ill-defined nodular lesions measuring 9.4 × 8.0 × 9.8 mm in the left lobe and 1.6 × 0.8 × 1.3 cm in the isthmus within a diffusely heterogeneous multinodular thyroid. This was significantly different from a thyroid ultrasound 3 years prior which did not have nodules present (Fig 1). Pathology from the fine needle aspiration biopsy (FNAB) of the nodules was benign. Genetic testing was performed due to the presence of a first-degree relative with CS and revealed heterozygosity for the p.S1701 pathogenic mutation for the phosphatase tensin homolog (PTEN) gene.

**Figure 2:**
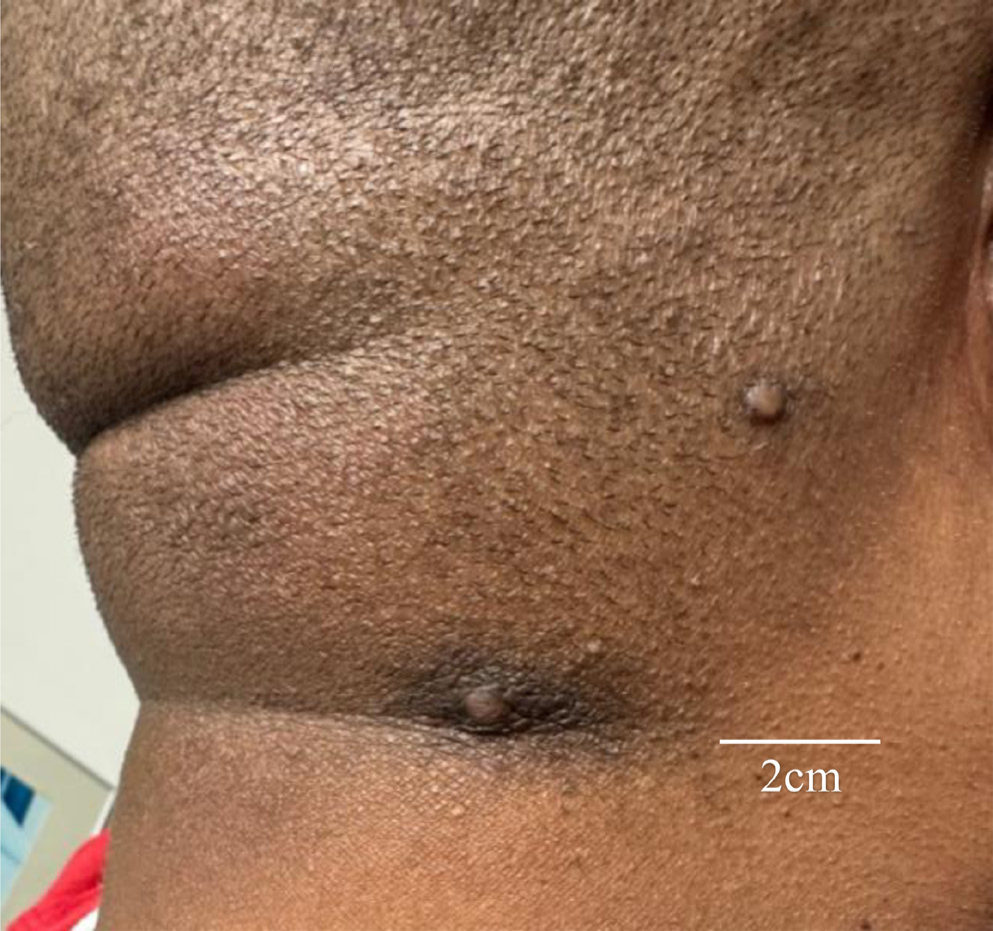
Two trichilemmomas present on the right lateral aspect of the patient’s neck.

Furthermore, patient 2 had a previous thyroidectomy because of follicular thyroid cancer 4 years prior to presentation followed by radioactive iodine therapy. He also had various trichilemmomas on his hands and neck (Fig. 2) for which dermatology recommended a colonoscopy. A screening colonoscopy revealed numerous colon polyps. The culmination of these factors led to an increased suspicion of CS. The patient underwent genetic testing revealing heterozygosity for the* c.723dupT* pathogenic PTEN mutation as well as the *p.S598L* mutation. His thyroid function tests showed a thyrotropin of 0.18 μIU/mL (0.450–4.50 μIU/mL) (0.18 mIU/L) and free thyroxine of 1.27 ng/dL (0.82–1.77 ng/dL) (8.81 pmol/L) while on levothyroxine 175 µg daily at this time. Thyroglobulin level and thyroglobulin antibody were <1.0 IU/mL.

## Treatment

Patient 1 underwent a total thyroidectomy. Surgical pathology showed a left 1.4 cm minimally invasive follicular carcinoma with angioinvasion and a right 0.4 cm papillary microcarcinoma without lymph node metastasis. The pathologic staging was pT1bpN0. Immunohistochemistry was positive for CD56, HBME1, and CK19.

After patient 2’s positive diagnosis, genetic screening of first-degree relatives was recommended. A review of colonic polyp pathology showed all polyps were consistent with chronic hyperplastic changes.

## Outcome and follow-up

Three weeks after patient 1’s surgery, her thyrotropin was 14.5 μIU/mL (0.450–4.50 μIU/mL) (14.5 mIU/L) and free thyroxine was 1.08 ng/dL (0.82–1.77 ng/dL) (7.50 pmol/L), hence her levothyroxine was increased to 125 µg daily in an effort to suppress her TSH. Her baseline thyroglobulin level was <0.1 ng/mL (SI: <0.1 µg/L) and thyroglobulin antibodies ≤4 IU/mL (SI: <4 kIU/L). Postoperative thyroid ultrasound showed no evidence of residual thyroid tissue. Subsequent thyroid ultrasounds 1 and 2 years post surgery continued to be negative for thyroid tissue or lymph node changes. The plan was to continue surveillance with annual thyroid ultrasounds and thyroid function tests. Patient 1 received genetic post-test counseling to discuss need for increased cancer surveillance.

Patient no. 2 was referred to general surgery for a colectomy. At the time of this publication, the patient had not yet undergone colectomy.

## Discussion

CS is a rare autosomal dominant condition caused by a mutation of the PTEN, a tumor suppressor gene (1). The loss of gene function leads to a higher prevalence of various malignancies (1, 2, 3, 4, 5).

The most common initial presentation appears as mucocutaneous lesions with a prevalence rate of up to 90% in patients with CS (1). One type of mucocutaneous lesion is trichilemmomas, which are wart-like, skin-colored papules with a slightly rough texture on the skin surface (1). Thyroid disease is the second most reported manifestation affecting half to two-thirds of patients with Cowden syndrome (3, 6). Thyroidal manifestations can include multinodular goiter, Hashimoto thyroiditis, and thyroid cancer (6, 7). As previously stated, patients are 70 times more likely to experience medullary thyroid cancer (7). The median age at cancer diagnosis in patients with CS was 35 years old (3). Breast, endometrial, renal, and colorectal cancers are also more prevalent with an incidence of 91%, 48%, 30%, and 17%, respectively, making early diagnosis critical (3).

Diagnosis can be aided by using a combination of major and minor criteria: any two major criteria with or without a minor criterion; one major and two minor criteria; or three minor criteria (Table 1) (1). Follow-up genetic testing should be obtained (1). Again, cancer surveillance should be performed for those with positive genetic testing and those with negative genetic testing but meet clinic diagnostic criteria (1). Management focuses on close cancer screening with special attention to the thyroid and breast. Screening should begin at the age of 18 years old or 5 years before the age of diagnosis of a family member (3). A colonoscopy should be performed at 35 years of age and consideration of renal ultrasound if there is concern (3).

**Table 1:** Revised PTEN hamartoma tumor syndrome clinical diagnostic criteria

Major criteria Breast cancer Endometrial cancer (epithelial) Thyroid cancer (follicular) Gastrointestinal hamartomas Lhermitte–Duclos disease (adult) Macrocephaly Macular pigmentation of the glans penis Multiple mucocutaneous lesions (any of the following) 1. Multiple trichilemmomas (≥3, at least one biopsy proven) 2. Acral keratoses (≥3 palmoplantar keratotic pits and/or acral hyperkeratotic papules) 3. Mucocutaneous neuromas (≥3) 4. Oral papillomas (≥3)
Minor criteria Autism spectrum disorder Colon cancer Esophageal glycogenic acanthosis (≥3) Lipomas (≥3) Mental retardation (i.e. IQ ≤75) Renal cell carcinoma Testicular lipomatosis Thyroid cancer (papillary or follicular variant of papillary) Thyroid structural lesions (adenomas, multinodular goiter) Vascular anomalies (including multiple intracranial developmental venous anomalies)
Operational diagnosis in an individual (either of the following) 1. Three or more major criteria, but one must include macrocephaly, Lhermitte–Duclos disease, or gastrointestinal hamartomas 2. Two major and three minor criteria
Operational diagnosis in a family where one individual meets revised PTEN hamartoma tumor syndrome clinical diagnosis criteria or has a *PTEN* mutation (any one of the following) 1. Any two major criteria with or without minor criteria 2. One major and two minor criteria 3. Three minor criteria

Although the FNAB did not indicate malignancy in patient 1, her family history, skin manifestations, multinodular goiter, and autism met the clinical diagnostic criteria for CS. Genetic testing confirmed the suspicion. Genetic pre-test counseling involves discussion of the appropriateness of testing, the ideal age to test, and the psychosocial implications of testing. Genetic post-test counseling includes tailoring their increased risk of various cancers in combination with their family history, appropriate specialist referrals, and implications on family members. The high prevalence of thyroid cancer and rapid changes seen on thyroid ultrasound prompted the decision to pursue thyroidectomy. Treatment of thyroid cancer follows the sporadic form. Patient no. 2 merely had hyperplastic changes, but his genetic mutation put him at high risk of eventual cancerous conversion. The constellation of medullary thyroid cancer, trichilemmomas, and multiple gastrointestinal polyps met the clinical diagnostic criteria for CS.

Endocrinologists are frequently requested to consult and manage patients with thyroid cancer. It is important that CS should be considered in thyroid cancer patients with a strong family history of cancer and the classic mucocutaneous lesions of CS. Early diagnosis and genetic counseling are essential for the treatment of these patients and for family members.

## Declaration of interest

The authors declare that there is no conflict of interest that could be perceived as prejudicing the impartiality of the study reported.

## Funding

This work did not receive any specific grant from any funding agency in the public, commercial, or not-for-profit sector.

## Patient consent

Written informed consent for publication of their clinical details and/or clinical images was obtained from the patients.

## Author contribution statement

SP composed the manuscript, literature review, provided figures and tables, and conducted pathology review. DJ was responsible for interpretation of data, revision of content, and final approval of the version published. All authors contributed to the article and approved the submitted version.
